# Cyclic-AMP and bacterial cyclic-AMP receptor proteins revisited: adaptation for different ecological niches^☆^

**DOI:** 10.1016/j.mib.2014.01.003

**Published:** 2014-04

**Authors:** Jeffrey Green, Melanie R Stapleton, Laura J Smith, Peter J Artymiuk, Christina Kahramanoglou, Debbie M Hunt, Roger S Buxton

**Affiliations:** 1 The Krebs Institute, Department of Molecular Biology and Biotechnology, University of Sheffield, Sheffield S10 2TN, UK; 2 Division of Mycobacterial Research, MRC National Institute for Medical Research, Mill Hill, London NW7 1AA, UK

## Abstract

*Escherichia coli* cyclic-AMP receptor protein (CRP) represents one of the paradigms of bacterial gene regulation. Yet despite decades of intensive study, new information continues to emerge that prompts reassessment of this classic regulatory system. Moreover, in recent years CRPs from several other bacterial species have been characterized, allowing the general applicability of the CRP paradigm to be tested. Here the properties of the *E. coli*, *Mycobacterium tuberculosis* and *Pseudomonas putida* CRPs are considered in the context of the ecological niches occupied by these bacteria. It appears that the cyclic-AMP-CRP regulatory system has been adapted to respond to distinct external and internal inputs across a broad sensitivity range that is, at least in part, determined by bacterial lifestyles.

## Highlights

• *E. coli* cyclic-AMP receptor protein (CRP) is a paradigm of gene regulation.
• Comparison of CRPs reveals differences in their affinity of cAMP.
• A range of dependency on cAMP for DNA-binding exists.
• CRPs have adapted to function in the specific niches occupied by the bacteria.

**Current Opinion in Microbiology** 2014, **18**:1–7

This review comes from a themed issue on **Cell regulation**

Edited by **Cecília Maria Arraiano** and **Gregory M Cook**

For a complete overview see the Issue and the Editorial

Available online 7th February 2014

1369-5274/$ – see front matter, © 2014 The Authors. Published by Elsevier Ltd. All rights reserved.

**http://dx.doi.org/10.1016/j.mib.2014.01.003**

## The *Escherichia coli* paradigm

The cyclic-AMP receptor protein (CRP) and its effector cyclic-AMP (cAMP) were discovered in *E. coli* during investigations to explain the phenomenon of diauxic growth more than 40 years ago. Since then, *E. coli* CRP–cAMP has become a paradigm of gene regulation, providing insights into signal perception and transduction, DNA recognition by regulatory proteins, regulator–polymerase interactions and promoter architecture [1].

The formation of the second messenger cAMP from ATP is catalyzed by a group of enzymes known as adenylyl cyclases. These enzymes are classified into six groups based on their primary structures. *E. coli* possesses a single Class I adenylyl cyclase (Cya) whose activity is controlled by glucose availability, such that growth at micromolar concentrations of glucose increases intracellular cAMP concentrations ∼10-fold (∼20 μm to ∼180 μm) compared to excess glucose conditions [2]. The consensus view has been that when the preferred carbon source glucose is available, it is transported into the cell by the glucose phosphotransferase system (PTS) and glucose enters the cytoplasm as glucose-6-phosphate [3,4]. The phosphorylation state of the PTS thus acts as a reporter of glucose availability — the phosphorylation state of the PTS is lower when the glucose transporter is saturated, whereas when glucose is absent, phosphorylated PTS proteins accumulate. The phosphorylated PTS interacts with Cya to enhance adenylyl cyclase activity [3]. Thus, when the bacteria are glucose-starved, the intracellular cAMP concentration increases as a consequence of the altered phosphorylation state of the PTS; however this is difficult to reconcile with observations that the glucose PTS is still saturated when intracellular cAMP concentrations increase [2]. Consequently, it has been suggested that cAMP concentrations increase in response to low energy charge, such that when ATP is low, cAMP is high, promoting catabolism and inhibiting anabolism by CRP–cAMP-mediated gene regulation to bridge the perceived energy deficit [5].

Degradation of cAMP is mediated by a phosphodiesterase, CpdA, but this enzyme has a rather high *K*_m_ for cAMP (∼500 μm) relative to the concentration of cAMP in the cell, and a *cpdA* mutant exhibited only a twofold increase in intracellular cAMP concentration [6]. Consequently, the observation that cAMP is often found extracellularly (0.03–0.5 μm) led to the finding that *E. coli* can quench intracellular levels of cAMP by TolC-mediated efflux, although the cAMP transporter(s) that links to the outer-membrane pore TolC has not yet been identified [7^•^].

**Figure 1:**
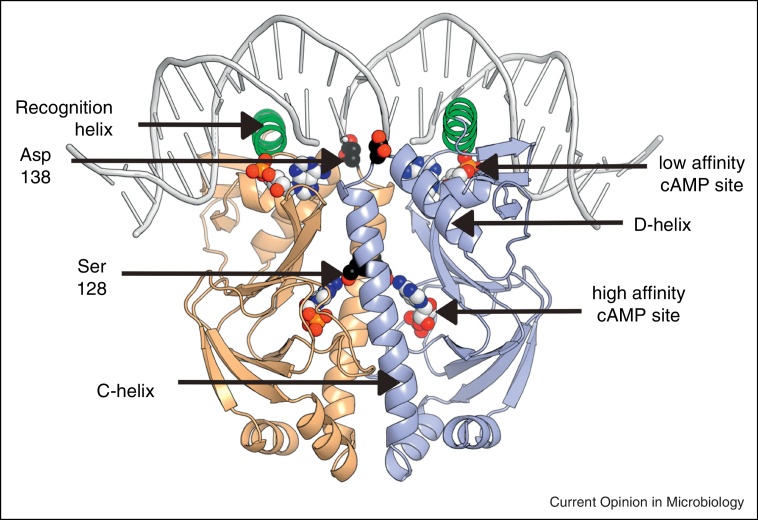
Relevant structural features of the *E. coli* CRP–cAMP–DNA complex. The CRP dimer (one protomer in brown, the second in blue) is shown in cartoon representation with the DNA-recognition helices highlighted in green. The locations of the C-helices at the dimer interface, the D-helices of the DNA-binding domain and the key residues Ser-128 and Asp-138 are indicated. Cyclic-AMP molecules bound in the *anti*-conformation at the higher affinity sites in the sensory domain and in the *syn*-conformation at the low affinity sites close to the DNA are shown in a ‘space-fill’ representation. DNA is shown as a pale gray ribbon. The diagram was constructed using Pymol [34].

Changes in intracellular cAMP concentration are perceived by the transcription factor CRP. CRP is a homodimer in which each subunit possesses three major structural features (Figure 1). The N-terminal region houses the high-affinity cAMP-binding domain and the C-terminal region consists of a DNA-binding domain with a canonical helix-turn-helix motif. These two domains are connected by a long helix (C-helix) that forms a coiled-coil at the dimer interface and a short linker followed by another helix (D-helix) (Figure 1). Cyclic-AMP binding to the sensory domain is initially exothermic (Δ*H*_1_ = −16.3 kJ mol^−1^; Δ*S*_1_ = 41 J K^−1^ mol^−1^) followed by an endothermic phase (Δ*H*_2_ = 25.1 kJ mol^−1^; Δ*S*_2_ = 176 J K^−1^ mol^−1^) and cAMP interactions with the two protomers that make up the CRP dimer are cooperative (Δ*G*_2_ − Δ*G*_1_ = 2.7 kJ mol^−1^) with binding constants of 8 × 10^4^ m^−1^ for site 1 and 6 × 10^4^ m^−1^ for site 2 [8^•^].

**Figure 2:**
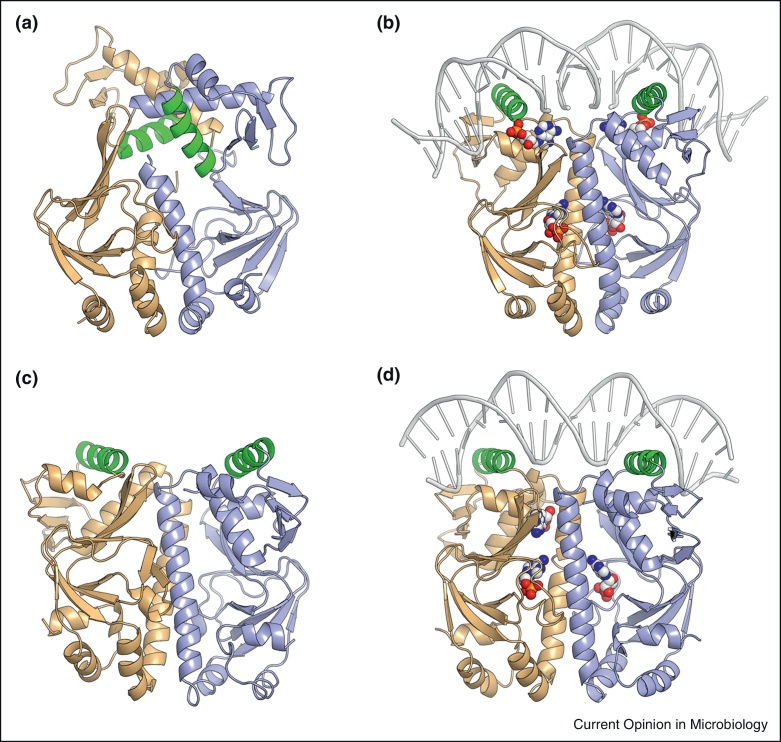
X-ray crystal structures of *Escherichia coli* and *Mycobacterium tuberculosis* CRPs in the absence and presence of cAMP. **(a)***E. coli* apo-CRP (PDB ID 3HIF). **(b)***E. coli* cAMP–CRP (PDB ID 2CGP). **(c)***M. tuberculosis* apo-Rv3676 (PDB ID 3D0S). **(d)***M. tuberculosis* cAMP-Rv3676 (PDB ID 3MZH). The diagram was constructed and the features are highlighted as described in the legend to Figure 1.

In the apo-CRP dimer, the two DNA-binding domains interact to form a rigid body in which the DNA-recognition helices are buried such that they cannot interact with DNA [9]. Binding of cAMP to CRP initiates structural rearrangements about a ‘hinge’ region allowing the DNA-binding domains to relocate relative to the cAMP-binding domain in a process mediated through hydrogen bonds between the N(6) position of adenosine with Ser-128 of the dimerization helices (C-helices; Figure 1) [9,10]. This allosteric conversion critically involves extension of the C-helices by six residues and shortening of the D-helices by four residues, such that Asp138 becomes the N-terminal capping residue of the D-helix in CRP–cAMP (Figure 1), but is an internal part of the longer D-helix in apo-CRP (Figure 2a and b). The helix-capping propensity of residue 138 is correlated to the degree of co-operative cAMP-binding and hence this property of Asp138 is a key feature of the interdomain conformational changes that modulate the apo-CRP ↔ CRP–cAMP equilibrium [8^•^]. NMR spectroscopy and thermodynamic analyses of several CRP variants revealed how changes in conformational entropy modulate DNA-binding activity [11^••^]. These structural rearrangements expose the DNA-recognition helices (highlighted in green in Figure 1) such that they are able to participate in sequence specific (consensus sequence TGTGAnnnnnnTCACA) binding at two adjacent major grooves of DNA (Figure 2b) [12].

Cyclic-AMP binding has a biphasic effect on site-specific DNA-binding by CRP. High-affinity binding of cAMP in the *anti*-conformation at the sensory domains of the CRP subunits (1:1 cAMP/CRP protomer) enhances DNA-binding ∼1000-fold. This is followed by decreased DNA-binding when cAMP in the *syn*-conformation interacts with weak binding sites formed by components of the helix-turn-helix, a β-hairpin from the regulatory domain and DNA (2:1 cAMP/CRP protomer) [13]. However, proof that cAMP-binding to these low affinity sites is of physiological significance has not yet been provided.

CRP–cAMP binding to intergenic CRP sites is associated with classical gene regulation [14,15^•^]; however, it is now recognized that CRP–cAMP also binds at many intragenic sites where it is thought to fulfill a role as a chromosome organizer or nucleoid associated protein (NAP) [14]. Genome SELEX screening identified at least 254 CRP-binding sites across the *E. coli* genome and because CRP is capable of controlling expression of divergent promoters from a single binding site it is estimated that CRP–cAMP directly controls a minimum of 378 promoters, and perhaps >500 genes in *E. coli* [15^•^]. Amongst these operons, CRP–cAMP acts as the ‘master’ regulator for 70 ‘slave’ transcription factors further expanding the profound influence of CRP on global gene expression in *E. coli*, in which it plays a key role in managing catabolism, including the transport of substrates, glycolysis, the Krebs cycle and aerobic respiration [15^•^,16].

## Variation 1 — *Mycobacterium tuberculosis* CRP, Rv3676, a regulator evolved to operate at high cAMP concentrations?

Unlike *E. coli*, which has only one adenylyl cyclase, *M. tuberculosis* H37Rv possesses at least 16 Class III adenylyl cyclase-like proteins, including soluble and membrane-associated multidomain proteins, suggesting that their catalytic activities (10 of the 16 have been shown to act as adenylyl cyclases) can be regulated by extracellular and/or intracellular signals, reviewed by Chakraborti [17]. Accordingly, adenylyl cyclase activity of *M. tuberculosis* is affected by pH, CO_2_, and fatty acids. It has long been recognized that mycobacteria secrete cAMP, but it is only more recently that the capacity to intoxicate macrophages with cAMP has been recognized as a contributor to virulence [18,19]. Thus, the synthesis (in particular by Rv0386) and secretion of cAMP are central features of *M. tuberculosis* pathogenesis and result in the bacterium being exposed to relatively high concentrations of cAMP; there are reports of intracellular concentration of cAMP as high as 4 mm for *M. tuberculosis* H37Rv and ∼3 mm for *Mycobacterium smegmatis*, which far exceed values reported for *E. coli* [20,21]. However, it is wise to offer a note of caution here; because the different growth conditions and methods used to measure cAMP, it is difficult to make direct comparisons. Nevertheless, the important role that cAMP plays in tuberculosis pathogenesis exposes the need for careful investigation of both intracellular and extracellular cAMP concentrations using modern approaches.

The relatively high concentrations of cAMP reported for mycobacteria are consistent with the presence of multiple adenylyl cyclases but of only one cAMP phosphodiesterase (Rv0805) in *M. tuberculosis* H37Rv. Moreover, the cAMP phosphodiesterase activity of Rv0805 is poor, and like its *E. coli* counterpart, it has a relatively high *K*_m_ for cAMP (∼150 μm) [22]. This rather poor *in vitro* activity is reflected *in vivo*, where overproduction of Rv0805 resulted in only a ∼30% decrease in cAMP (a ∼90% decrease was observed for overproduction of CpdA in *E. coli*), perhaps indicating alternative roles for this enzyme, which also possesses the ability to hydrolyze a range of cNMP and linear phosphodiesters [23^•^]. In the light of these data, it has been suggested that intracellular cAMP levels might be controlled by excretion rather than conversion to AMP but, as is the case for *E. coli*, there is a need to establish the mechanism(s) of cAMP secretion and how this might be regulated.

The *M. tuberculosis* CRP (Rv3676; 32% amino acid identity to *E. coli* CRP over 189 amino acids, including four of the six key cAMP-interacting residues in the sensory domain of *E. coli* CRP; Table 1) differs from the *E. coli* paradigm at several levels. The Rv3676 homodimer exhibits relatively weak (*K*_b_ = 1.7 × 10^4^ m^−1^) binding of cAMP to two independent sites (1:1 cAMP/protomer). Furthermore, cAMP-binding is endothermic (Δ*H* = 30.7 kJ mol^−1^; Δ*S* = 183 J K^−1^ mol^−1^; Δ*G* = −23.7 kJ mol^−1^) and thus binding is entropically driven [24]. The independent nature of cAMP-binding to Rv3676 compared to *E. coli* CRP was accounted for by the replacement of a single amino acid residue (Ser-128 of CRP, which is required for the dramatic conformational changes that occur upon cAMP-binding, is replaced by Asn in Rv3676) that has the effect of reorganizing a hydrogen-bonding network involving cAMP such that the cAMP-binding sites in Rv3676 are uncoupled [24]. It has been argued that the relatively weak and independent binding of cAMP at the sensory domain of Rv3676 has evolved to allow the protein to be at least partially cAMP-responsive against the background of high cAMP concentrations required to intoxicate the host during infection.

The crystal structures of apo-Rv3676 and Rv3676-cAMP reveal that cAMP-binding is associated with much less dramatic structural rearrangements than those observed for *E. coli* CRP [25–27] (Figure 2c and d). The major alteration that occurs upon cAMP-binding is weakening of the interactions between the DNA-binding and sensory domains, resulting in increased spatial freedom of the DNA-binding domain that is apparently sufficient to permit binding to target DNA sequences by an induced fit mechanism [27].

Consistent with the relatively mild structural rearrangements that occur upon cAMP-binding by Rv3676, the formation of the Rv3676–cAMP complex has a relatively small effect (∼2-fold) on DNA-binding to a consensus sequence that is very similar to that of *E. coli* (GTGnnAnnnnnCACA) [28]. Furthermore, unlike *E. coli* CRP, apo-Rv3676 is capable of site-specific DNA-binding and transcription regulation [24]. These observations are consistent with the limited overlap between genes dysregulated in the *crp* mutant and those affected by Rv0805 overproduction [23^•^] and suggests that the primary role of Rv0805 might not be to act as a cAMP phosphodiesterase and/or that Rv3676 can significantly influence gene expression without the need to bind cAMP.

Like *E. coli* CRP, *M. tuberculosis* Rv3676 is a global regulator but, perhaps unsurprisingly in the context of the very different lifestyles of these two bacteria, the corresponding CRP regulons differ. Thus Rv3676 appears to be involved in regulating the transition between replicating and nonreplicating states by exerting influence over virulence-critical functions, including phthiocerol dimycocerosate (DIM) synthesis, resuscitation promoting factors, the ESX-1 type VII secretion system, carbon metabolism, energy conservation and ‘slave’ transcription factors, such as the nitric oxide-responsive regulator WhiB1 [28,29]. This degree of control over the transcriptome is consistent with the attenuated state of the *M. tuberculosis crp* mutant in models of infection [28].

## Variation 2 — *Pseudomonas putida* CRP, PP**_**0424, a regulator evolved to operate at low cAMP concentrations?

*P. putida* possesses a CyaA-type adenylyl cyclase capable of cAMP synthesis (and there is also a second protein PP_5187 annotated as an adenylyl cyclase), but nevertheless cAMP concentrations are below the level of detection in bioassays [30^•^]. *P. putida* KT2440 possesses a cAMP phosphodiesterase (PP_4917) equivalent to the *E. coli* CpdA protein. Thus, the very low levels of cAMP in *P. putida* could arise from poor synthesis or rapid degradation, but based on complementation experiments with *E. coli cya* mutants the former is the more likely. Thus, *P. putida* seems to represent the opposite end of the ‘cAMP spectrum’ to *M. tuberculosis*. The *P. putida* CRP is 63% amino acid identical to *E. coli* CRP over 208 amino acids, including five out of the six cAMP-interacting residues in the sensory domain of *E. coli* CRP — interestingly the mismatch is again located at the position equivalent to 128 in *E. coli* CRP (Table 1). Consistent with the very low concentrations of cAMP, the *P. putida* CRP exhibits very high affinity (*K*_b_ = 4.4 × 10^7^ m^−1^) binding of cAMP to two independent sites (1:1 cAMP/protomer). Furthermore, cAMP-binding is exothermic (Δ*H* = −25 kJ mol^−1^; Δ*S* = 63 J K^−1^ mol^−1^; Δ*G* = −10.5 kJ mol^−1^), and binding is both enthalpy and entropy driven [31^••^]. Although detailed structural information is not yet available, this hypersensitive binding of cAMP invokes large conformational changes that can be detected by size exclusion chromatography and result in enhanced DNA-binding to a typical CRP inverted repeat sequence by >10-fold [31^••^].

Although *P. putida* exhibits catabolite repression, this behavior is not mediated by CRP–cAMP, as a *crp* mutant was unaffected in its ability to utilize a full range of sugars as carbon sources [32]. Rather, CRP–cAMP appears to control the utilization of l-phenylalanine and of various dipeptides as nitrogen sources in *P. putida* [30^•^,33]. The full range of genes regulated by CRP in *P. putida* has not been established but a conservative analysis of the genome sequence for likely binding sites indicates that >30 genes might be regulatory targets, few of which appear to have a metabolic role [30^•^]. These observations led to the suggestion that *P. putida* and *M. tuberculosis* CRPs have evolved to control different biological processes compared to the *E. coli* paradigm, a possible example of regulatory exaptation [30^•^].

## Perspectives and outstanding questions

In recent years many aspects of the cAMP-signaling CRP-regulatory paradigm that has emerged from intensive studies of catabolite repression in *E. coli* have come under closer scrutiny. Almost every step from the relationship between the activity of the glucose PTS and cAMP synthesis to the role of cAMP–CRP in gene regulation and chromosome organization has been reassessed. The picture that is emerging is one in which intracellular and extracellular cAMP concentrations are modulated by adenylyl cyclases, phosphodiesterases and cAMP efflux systems some of which respond to external and/or internal signals. Changes in intracellular cAMP concentration are perceived by CRP proteins that react with different sensitivities related to the niches occupied by the bacteria. Thus, the pathogen *M. tuberculosis* Rv3676 is a low sensitivity CRP evolved to maintain some degree of responsiveness at the high cAMP concentrations used to intoxicate host macrophages; the commensal enteric bacterium *E. coli* possesses a mid-sensitivity CRP to regulate catabolite repression and chromosome structure, probably in response to energy charge; and the soil bacterium *P. putida* has a hypersensitive CRP, reflecting the very low concentrations of cAMP produced by this bacterium. Nevertheless, despite decades of study there are still many outstanding questions that need to be addressed to complete our understanding of cAMP-signaling in *E. coli* and other bacteria. For example, at the root of cAMP-mediated signaling in bacteria is the ability to synthesize and degrade cAMP in response to environmental and metabolic signals. To place investigations of cAMP-signaling on a sound footing there is a need to apply the latest metabolite quenching, extraction and analysis techniques to accurately measure intracellular and extracellular cAMP concentrations under diverse growth conditions.

Further characterization of the phosphodiesterases involved in cAMP degradation and the processes required for cAMP excretion is required. Signal-dependent synthesis of cAMP is only one component in controlling bacterial responses to this second messenger; there have to be mechanisms for removing cAMP from the system. It appears that the cAMP phosphodiesterases are rather poor enzymes, leading to the suggestion that secretion of cAMP might be the major route to lowering cAMP in the cell. However, thus far no cAMP efflux systems have been identified beyond the recognition that TolC is involved in facilitating cAMP crossing the outer membrane of *E. coli*. Identifying cAMP secretion systems and defining their role in bacterial signaling and pathogenesis will fill a major deficit in our current knowledge.

It will be informative to seek physiological and evolutionary explanations for the differences in cAMP-binding to the regulatory CRP proteins in different bacteria, in particular, establishing whether CRPs, like that of *M. tuberculosis*, which exhibits only a mild enhancement in DNA-binding in response to cAMP, control cAMP-independent and cAMP-dependent regulons. Similarly, mechanistic explanations for the extremely avid cAMP-binding by CRPs, as exemplified by the *P. putida* CRP, should be sought. At present it appears that responding to the second messenger cAMP allows CRP to be co-opted to control different regulons in bacteria that occupy distinct niches. Thus, because cAMP intoxication of the host is an important component of *M. tuberculosis* pathogenicity, its CRP has become desensitized to cAMP, whereas the CRP of the soil bacterium *P. putida* has become hypersensitive to cAMP. Hence through a common mechanism of CRP-mediated RNA polymerase recruitment and signal-dependent cAMP synthesis/degradation (e.g. glucose availability in *E. coli*; pH and other virulence-related signals in *M. tuberculosis*; unknown signals possibly related to the utilization of aromatic amino acids and nitrogen sources by *P. putida*), CRP has been co-opted to control distinct regulons according to the particular niches occupied by the bacteria. Developing a mechanistic framework that accounts for the shifts in cAMP-binding affinities observed in different CRPs would then allow questions about the physiological roles of cAMP–CRP complexes with alternative stoichiometries to be addressed.

## References and recommended reading

Papers of particular interest, published within the period of review, have been highlighted as:

• of special interest
•• of outstanding interest

## Figures and Tables

**Table 1:** Comparison of features of cAMP-signaling in three bacteria

Bacterium	*M. tuberculosis*	*E. coli*	*P. putida*
Niche	Lung macrophage	Mammalian intestine	Soil
Number of adenylyl cyclases	16	1	2
Intracellular cAMP concentrations	High	Moderate	Low
CRP	Rv3676	CRP	PP_0424
cAMP–CRP interactions	Independent binding	Cooperative binding	Independent binding
*K*_D_ for cAMP	∼60 μm	∼13–16 μm	∼23 nm
Motif for cAMP interaction^a^	E…TS…R…T**N**	E…RS…R…T**S**	E…RS…R…T**T**
Number of phosphodiesterases	1	1	1
Number of chromosomal binding sites^b^	>70	>378	>30

a Amino acids involved in direct interaction with cAMP in *E. coli* CRP as single letter code with dots (…) representing intervening regions of various lengths. The amino acid at the position equivalent to Ser-128 in *E. coli* CRP that makes a cross-subunit contact with cAMP is shown in bold font.

b The binding site numbers represent: matches to the Rv3676 consensus sequence identified by Rickman *et al.* [28] in the *M. tuberculosis* H37Rv genome sequence; *E. coli* CRP-binding sites suggested by Shimada *et al.* [15^•^] following genomic SELEX analysis; and a minimum value based on interrogation of the *P. putida* KT2440 genome sequence by the *E. coli* CRP-binding site consensus [30^•^].
